# Oral Feeding of NICU Infants: A Global Survey of Current Practices and the Potential of Cold Milk Feeding Intervention

**DOI:** 10.3390/nu17142289

**Published:** 2025-07-10

**Authors:** Zeyar T. Htun, Louisa Ferrara-Gonzalez, Ranjith Kamity, Nazeeh Hanna

**Affiliations:** Division of Neonatology, Department of Pediatrics, NYU Langone Hospital—Long Island, NYU Grossman Long Island School of Medicine, Mineola, NY 11501, USA; zeyar.htun@nyulangone.org (Z.T.H.); louisa.ferrara123@gmail.com (L.F.-G.); ranjith.kamity@nyulangone.org (R.K.)

**Keywords:** dysphagia, cold milk, oral feeding, preterm, NICU, neonatal, infant

## Abstract

**Background/Objectives**: Infants admitted to neonatal intensive care units (NICUs) face challenges in achieving successful oral feedings. During oral feedings, these infants commonly present with suck–swallow–breathe incoordination, with approximately 30% developing dysphagia, leading to feeding aversion, prolonged hospitalization, and increased parental stress. Cold liquid feeding has demonstrated benefits in improving feeding safety in adults with dysphagia; however, its application in neonates is relatively limited. This study aimed to examine global neonatal feeding practices, with a specific emphasis on cold milk feeding as an intervention for dysphagia. **Methods**: A cross-sectional global electronic survey was distributed via professional society listservs and closed online professional group forums targeting neonatal providers and feeding therapists from June 2023 to June 2024. The survey assessed institutional feeding protocols, oral feeding practices, and the use of cold milk for infants with dysphagia. Responses were analyzed descriptively. **Results**: A total of 210 complete responses were received from level IV (51%), level III (42%), and level II (5%) NICUs. While 30% of the respondents were aware of cold milk feeding as a dysphagia intervention, only 15% of the total respondents reported using it in practice. Among the 32 institutions implementing cold milk practices, only one had an established protocol. Additionally, 72% reported having a feeding protocol in place, often incorporating cue-based tools. Most respondents (87.5%) did not allow oral feeding during nasal continuous positive airway pressure (nCPAP), whereas 78% permitted it during high-flow nasal cannula (HFNC) support. **Conclusions**: Although the awareness of cold milk feeding in neonates is increasing, its implementation remains limited and lacks standardization. Significant variability exists in oral feeding practices, particularly regarding feeding during respiratory support. This underscores the need for further research and evidence-based guidelines to ensure safe and consistent care for preterm infants.

## 1. Introduction

Achieving successful oral feeding is one of the final milestones hospitalized infants must reach before safe discharge from neonatal intensive care units (NICUs). Oral feeding depends on the infant’s ability to appropriately coordinate sucking, swallowing, and breathing while protecting the airway. The sucking and swallowing skills start to develop in the womb as early as 12 to 13 weeks of gestational age but do not become more rhythmical and predictable until approximately 32–34 weeks [1]. Historically, infants born prematurely were generally introduced to oral feedings when they reached 32–34 weeks postmenstrual age (PMA) [2,3]. However, their ability to coordinate breathing at these early gestational ages is still neurologically underdeveloped, as a full-term infant would not have to do so until 40 weeks. Delays or persistent immaturity in this developmental process can impair coordination and increase the risk of dysphagia. Dysphagia is observed in approximately 30% of very low birth weight preterm infants [4]. Dysphagia, defined as difficulty swallowing, resulting in tracheal aspiration or laryngeal penetration, can lead to respiratory complications, increase the risk of feeding aversion, prolong hospitalization, and heighten parental stress. In the NICU, the current interventions for managing dysphagia include pacing, thickening enteral feeds (with cereals or commercial thickeners), using slow-flow bottle nipples, and positioning the infant in an elevated side-lying position [2,5,6,7,8]. While these first-line strategies may offer some benefit, their therapeutic effect is often limited. As a result, there is a need for additional interventions, and one novel approach in the neonatal population is the use of cold milk feeding.

Cold milk feeding has the potential to promote more efficient and safer swallowing. In adults with dysphagia secondary to stroke, cold liquid swallows have demonstrated therapeutic benefits [9,10]. Studies in both animal models and adults suggest that cold liquid stimulates sensory thermo-receptors in the pharyngeal region, enhancing the sensory input to the brain and improving the pharyngeal swallowing response [10,11]. We previously published a pilot study in preterm infants demonstrating that those fed cold liquid barium experienced a significant reduction in deep laryngeal penetration and tracheal aspiration events compared to those fed room-temperature barium under videofluoroscopy [12]. Our second, more recently published study confirmed the safety of feeding preterm infants cold milk at the bedside [13]. While emerging research is encouraging, understanding how to utilize cold milk as a management option for neonatal dysphagia remains undefined and lacks standardized guidelines.

The safety of initiating oral feeding in infants receiving high-flow nasal cannula (HFNC) or nasal continuous positive airway pressure (nCPAP) remains unclear, as current evidence is limited [14,15,16,17,18,19]. The potential for laryngeal penetration and tracheal aspiration, especially in infants with bronchopulmonary dysplasia (BPD), raises the risk of respiratory infections and warrants caution against aggressive oral feeding approaches [18]. Our previously published study indicates that oral feeding while on nCPAP is associated with increased aspiration and penetration [17].

Other studies suggest that deferring oral feeding can adversely affect the developmental trajectory of infants and young children due to prolonged stays in the intensive care unit [18,19]. However, two recent retrospective studies highlight that waiting for an infant to wean off their supports before starting oral feeds carries no significant risks [14,20]. Massarelli et al. (2025) examined the impact of extended CPAP use on oral feeding and length of stay in preterm infants [20]. In this cohort, oral feeding was not permitted while on CPAP, and prolonged CPAP use was associated with increased postmenstrual age (PMA) at achievement of full oral feeds. However, the difference did not translate into a clinically significant increase in length of stay. These findings were corroborated in our previously published study, which found that delaying oral feeding until preterm infants were off nCPAP did not result in feeding-related morbidities and did not delay home discharge [14]. Furthermore, there was a positive correlation between the duration of oral feeding while on nCPAP and the time spent on respiratory support.

In a recent survey, speech–language therapists demonstrated inconsistent approaches when managing oral feeding in infants on CPAP or HFNC [21]. While there are shared strategies for assessing feeding readiness and observing tolerance, the absence of clear guidelines and standardized protocols means decisions often rely heavily on clinical experience. This underscores a significant gap in evidence-based practice that warrants further investigation.

There is a notable lack of research on dysphagia in hospitalized term and preterm infants, which has limited the development of standardized evidence-based approaches to this common clinical challenge. Therefore, we conducted a global online survey targeting neonatal intensive care units to gain insight into the current oral feeding practices for NICU infants, with a particular emphasis on the use of cold milk feeding.

The objectives of this study were to

(1) Evaluate the prevalence of cold milk feeding as a therapeutic strategy for managing dysphagia in hospitalized infants.
(2) Identify the gestational age at which oral feeding is typically initiated for those born preterm.
(3) Investigate the types of noninvasive respiratory support, such as nCPAP and HFNC, that are permitted during oral feeding in hospitalized infants.

## 2. Methods

### 2.1. Study Design

This study utilized a cross-sectional survey design to assess oral feeding practices, including the use of cold milk, across neonatal intensive care units (NICUs). The survey was distributed between June 2023 and June 2024. Survey reporting adhered to established guidelines for best practices in survey-based research [22].

### 2.2. Participants

Eligible participants included neonatal healthcare professionals involved in the care of preterm infants, such as neonatologists, neonatal fellows, advanced practice providers (APPs), and neonatal therapists (SLPs, OTs, PTs). Participation was voluntary and anonymous.

### 2.3. Survey Distribution Procedures

The survey was disseminated electronically using the REDCap platform at NYU Langone Health. Distribution was conducted via professional society listservs and closed online professional group forums, including the Section on Neonatal-Perinatal Medicine (SoNPM), American Speech-Language-Hearing Association (ASHA), National Association of Neonatal Therapists (NANT), Society for Ear Nose and Throat Advancement in Children (SENTAC), and American Occupational Therapy Association (AOTA). Responses were collected directly from individuals, not institutions or the societies themselves. Although the survey was distributed through professional society listservs, it was disseminated broadly without a centralized distribution list, and therefore the exact number of recipients could not be determined—precluding calculation of a formal response rate.

### 2.4. Survey Content

The survey included multiple-choice, Likert-scale, and free-text questions. It assessed

- The presence and type of oral feeding protocols;
- The timing of oral feeding initiation;
- The use of cold milk for dysphagia;
- The oral feeding practices during noninvasive respiratory support (nCPAP, HFNC).

A full copy of the survey is included in Supplementary Figure S1.

### 2.5. Data Analysis

Responses were exported from REDCap (Version 14.5.43) and analyzed using Microsoft Excel. Descriptive statistics (frequencies, percentages, and means) were used to summarize the data. Incomplete or implausible responses were excluded from analysis. No inferential statistics were applied, given the descriptive nature of this study.

### 2.6. Ethical Considerations

The NYU Institutional Review Board determined that the survey did not constitute human subject research and was exempt from review and informed consent requirements.

## 3. Results

A total of 212 responses were received globally; however, 73% originated from respondents within the United States, while 15% came from individuals outside the United States (Table 1 and Figure S2). Two responses were excluded due to incompleteness or insufficient data. Among the valid responses, 47% were submitted by attending neonatologists, 46% by neonatal therapists, and 8% by neonatal fellows or advanced practice providers. The survey captured responses from a range of NICU settings, including level IV NICUs (52%), level III NICUs (42%), and level II NICUs (5%) (Table 1).

### 3.1. Feeding Protocols and Clinical Practices

As shown in Table 1, out of 210 valid responses, 72% (152/210) of the participants reported that their NICU had an established feeding protocol. Among those, 43% (66/152) used the Infant-Driven Feeding Scale (IDFS), while 44% (67/152) relied on other cue-based methods, such as the Preterm Infant Breastfeeding Behavior Scale (PIBBS), Supporting Oral Feeding in Fragile Infants (SOFFI), or institution-specific protocols—structured tools used to guide feeding initiation and progression based on infant readiness cues. The remaining 13% (19/152) did not specify the method used.

### 3.2. Timing of Oral Feeding Initiation

The gestational age at which oral feeding was initiated varied across NICUs. Among the respondents, 36% reported initiating oral feeds at or before 33 weeks’ gestation, 43% at 34 weeks, and 9% at 35 weeks (Table 1). Overall, 70% of the respondents indicated that feeding readiness cues were used alongside gestational age to determine the appropriate timing for initiating oral feeding. These findings suggest that 33 to 34 weeks’ gestation is commonly considered an appropriate developmental window for introducing oral feeding in preterm infants.

### 3.3. Awareness and Implementation of Cold Milk Feeding

A total of 30% (64/210) of the respondents were aware of cold milk feeding as a potential intervention for neonatal dysphagia (Table 2). Of those aware, 50% (32/64) indicated that this practice was allowed at their institution. However, only 16% (32/210) of all respondents reported using cold milk feeding in clinical practice. Among those who practiced cold milk feeding, the intervention was typically implemented in term and preterm infants older than 35 weeks’ postmenstrual age (78%). Of those who were practicing cold milk feeding, 40% had been practicing for ≥5 years, 75% had not noticed any adverse events, most did not thicken cold milk (69%), and most did not discharge the infant home on cold milk (56%). Surprisingly, only one institution reported following a specific protocol for cold milk feeding. The comments received from the respondents provided a range of perspectives on the use of cold milk feeding, highlighting the perceived potential benefits and concerns (Table 3).

### 3.4. Oral Feeding While on Respiratory Support

When asked about oral feeding in the context of noninvasive respiratory support, 12% (26/210) of the respondents reported feeding infants while on nCPAP (Table 4). Among these, 73% (19/26) of the respondents stated that an nCPAP +5 or less was required before initiating oral feeding. HFNC was more commonly used with oral feeding, with 78% (164/210) of the respondents indicating that oral feeding was permitted while on HFNC. Of these, 87% (143/164) reported feeding infants at flow rates of 2 L per minute or less. The comments received from the respondents provided a range of perspectives on the use of respiratory support during oral feeding, highlighting the perceived potential concerns (Table 5).

## 4. Discussion

To the best of our knowledge, this is the first global survey distributed through professional society listservs and social media groups to assess cold milk feeding practices in NICUs. Our study captured responses from a wide range of international neonatal providers across level II, III, and IV NICUs. The results provide insight into evolving oral feeding practices, especially the adoption of oral feeding for infants while on respiratory support and the emerging but unstandardized use of cold milk feeding for neonatal dysphagia management.

### 4.1. Cue-Based Feeding in Preterm Infants

Historically, oral feeding in preterm infants was initiated based on gestational age and advanced using volume-driven models. This approach often overlooked an infant’s individual developmental readiness and led to potential adverse outcomes such as feeding aversion, poorer neurodevelopmental outcomes, and prolonged feeding difficulties up to 4 years of age [23]. Cue-based feedings are centered around recognizing infant-driven behavioral and physiological signs of feeding readiness rather than relying solely on gestational age or prescribed volumes. These cues typically include alertness, rooting, bringing hands to mouth, tone, and posture as well as stability in vital signs before and during feeding. Cue-based protocols are structured tools used to guide feeding initiation and progression based on infant readiness cues. Our findings show that 72% of respondents reported the use of an established feeding protocol, with the majority adopting cue-based frameworks such as the IDFS, SOFFI, PIBBS, or institution-specific models [24,25,26]. This reflects a significant increase compared to a 1994 survey by Siddell et al., where only 36% of units reported having any feeding protocol [27]. This trend suggests a growing commitment to structured and developmentally appropriate feeding practices. Although our survey suggests a general consensus around initiating oral feeding between 33 and 34 weeks’ postmenstrual age, the majority (70%) of providers incorporated feeding readiness cues. Cue-based protocols promote safer and more developmentally supportive feeding experiences and are increasingly recognized as best practices in preterm infant care. A recent systematic review found that cue-based feeding shortens the time to achieve full oral feeding, reduces the incidence of feeding intolerance, and promotes parental involvement in feeding behavior for preterm infants [28].

### 4.2. Cold Milk Feeding in the NICU: Emerging but Unstandardized

Cold milk feeding, while commonly used in adult dysphagia management, remains a novel intervention in the neonatal population. Cold liquids provide the sensory receptors within the pharynx increased sensory information, which triggers more efficient swallowing movements. Evidence supporting the use of cold milk in preterm infants remains limited but is growing. Several past studies have revealed good tolerance of cold feeding in term and preterm infants, with no significant adverse effects on the infants’ sleep patterns, vocalizations, motility, intake, feeding behavior, weight gain, temperature, frequency of regurgitation, or gastric emptying time [29,30,31,32]. Ferrara et al. (2018) [12] evaluated preterm infants with diagnosed dysphagia (mean gestational age at birth: 30 weeks + 2 days; postmenstrual age at time of study: 39 weeks). This was a within-subject crossover design conducted in a tertiary NICU setting [12]. The intervention was cold barium feeding compared to room-temperature barium during video-fluoroscopic swallow studies. Cold barium feeding led to a significant reduction in deep laryngeal penetration and tracheal aspiration events (from 71% to 26%). In a subsequent observational safety study by Ferrara-Gonzalez et al. (2025), cold milk feedings given to preterm infants with uncoordinated feeding patterns did not significantly affect body temperature, gastric tolerance, or mesenteric perfusion when compared to room-temperature milk [13]. Although the findings from these initial studies are promising, broader evidence remains limited in scope and generalizability. No randomized controlled trials have been conducted to assess long-term efficacy, safety, or developmental feeding outcomes. These limitations underscore the need for well-designed multicenter trials to establish standardized protocols and evaluate the clinical utility of cold milk feeding in routine NICU care.

In our survey, 30% of the respondents were aware of cold milk feeding, and 16% actively used it in clinical practice. Most of these institutions administered cold milk to infants ≥35 weeks PMA, typically in cases of persistent dysphagia unresponsive to standard interventions. Given that cold milk feeding remains an emerging and under-researched intervention in neonatal care, we aimed to capture the perceptions, clinical observations, and experiential insights of frontline providers. Respondents who used cold milk noted perceived benefits such as improved swallowing coordination, engagement during feeds, and enhanced sensory awareness—consistent with theories suggesting cold liquid may stimulate the thermo-receptors in the pharynx, triggering a stronger pharyngeal swallow reflex. While studies suggest the safety of this practice, several respondents expressed concerns regarding feeding intolerance, refusal, discomfort, and risk of hypothermia. These comments offer valuable context regarding current attitudes, perceived benefits, and concerns associated with the practice. While not intended to provide definitive scientific evidence, these qualitative data serve as an important starting point to identify knowledge gaps and inform the design of future prospective studies. Despite its potential, cold milk feeding lacks formal protocols and remains inconsistently applied. Only one institution in our survey had a defined protocol in place, underscoring the urgent need for further clinical research to establish safety, efficacy, and implementation guidelines.

### 4.3. Lack of Consensus on Feeding During Noninvasive Respiratory Support

Feeding preterm infants who require respiratory support presents another clinical challenge. In our survey, 12% of the respondents permitted oral feeding while infants were on nCPAP, typically under strict criteria such as PEEP ≤ 5 cm H_2_O. In contrast, 78% allowed oral feeding on HFNC, though most limited feeding to flow rates of ≤2 L/min. Concerns of adverse events surrounding oral feeding on nCPAP or HFNC include increased risk of aspiration, poor coordination, undue physiologic stress, and respiratory instability [33]. Our findings are consistent with prior survey data obtained by Canning et al. (2020), who reported wide variability in feeding practices and notable disagreement among providers within the same institution [16]. Canning et al. reported differing opinions on feeding safety across providers in the same institution, with 76% of SLPs, 40% of physicians, and 32% of nurses expressing conflicting views [16]. Similar to their work, our study illustrates the absence of a consensus on the safe parameters for initiating oral feeding during respiratory support, particularly with nCPAP. Our survey study continues to highlight the variability that exists in the absence of strong evidence reinforcing the need for prospective trials to guide standardization.

### 4.4. Strengths and Limitations

This survey is the first, to the best of our knowledge, to explore cold milk feeding practices across international NICUs. The strengths include a diverse respondent pool representing a broad range of NICU levels and professional roles, as well as a relatively high response volume. In addition, given the known variability in oral feeding approaches and the lack of standardized protocols in many units, individual-level responses were particularly valuable in reflecting real-world practices. Therefore, the survey was distributed via societies and not directly to specific NICUs to gain the perspectives of practicing clinicians directly involved in daily NICU care. Furthermore, the study design for gathering descriptive data allowed us to identify the trends in clinical adoption (e.g., the increasing use of cue-based feeding models) and the variations in practices across institutions, which is a necessary first step in developing consensus guidelines. Several limitations are also acknowledged. Multiple responses may have originated from the same institution, as institutional identifiers were not collected in accordance with IRB guidance. While this could reflect intra-unit variation, it limited our ability to assess practices at the institutional level. Additionally, the survey included general questions regarding preterm infants without stratifying by specific subpopulations that are at heightened risk for dysphagia such as very low birth weight (VLBW) or extremely low gestational age neonates (ELGANs), which may influence feeding decisions and limit generalizability. Although a more focused study is warranted to assess specific subpopulations, our findings remain clinically meaningful. The majority of responses were from level III and level IV NICUs—units that routinely care for VLBW and ELGAN populations. Additionally, we collected information on corrected gestational age at the initiation of oral feeding and on the use of cue-based readiness assessments—both of which are highly relevant to feeding approaches in this vulnerable group. Another limitation is that the reliance on self-reported practices may have introduced recall or institutional bias. Lastly, the over-representation of U.S.-based respondents (85%) may limit the generalizability of the findings to global practices.

## 5. Conclusions

The growing use of cue-based feeding in NICUs reflects a positive shift toward individualized, developmentally appropriate, and neuroprotective care. However, significant variability persists in oral feeding practices, particularly regarding cold milk use and feeding during noninvasive respiratory support. Cold milk feeding is starting to emerge as providing promising benefits based on provider-reported experiences, even though there are limited clinical evidence and a lack of standardized institutional protocols. We also found a discrepancy between clinical practice and available high-quality evidence for feeding on nCPAP or HFNC. Therefore, these findings underscore the urgent need for evidence-based, standardized guidelines to enhance safety, reduce variability, and improve outcomes. Future research should prioritize evaluating the safety and efficacy of cold milk feeding and feeding on noninvasive respiratory support as well as the impact of these feeding practices on short-term clinical outcomes, such as time to full oral feeds, length of stay, and risk of aspiration, as well as long-term outcomes, such as the presence of feeding disorders into childhood and other neurodevelopmental delays. Multicenter trials and collaborative efforts among neonatologists, neonatal therapists, and researchers are essential to establish consensus-driven protocols that support consistent, high-quality care for infants in the NICU.

## Figures and Tables

**Table 1:** Survey responses to specific questions asked. Data presented as N and %.

Characteristics	N (%)
**Role of responders (*n*= 210)**	
Neonatal Attending	98 (47%)
Neonatal Fellow/Advanced Practice Provider	95 (45%)
Neonatal Therapist (SLP, OT, PT)	16 (8%)
No Response	1 (0.5%)
**NICU level responses (*n* = 210)**	
Level II	11 (5%)
Level III	88 (42%)
Level IV	108 (52%)
No Response	3 (1%)
**Location of the practicing NICU (*n* = 210)**	
United States	153 (73%)
International	32 (15%)
No Response	25 (12%)
**Does your institution currently have an oral feeding protocol for preterm infants? (*n* = 210)**	
Yes	152 (72%)
No	58 (28%)
**If yes, what protocol do you follow (i.e., IDFS, cue-based feeding, etc.)? (*n* = 152)**	
IDF	64 (43%)
Cue based	67 (44%)
Mixed	12 (8%)
No response	7 (5%)
**What corrected gestational age do preterm infants begin oral feeding? (check all that apply) (*n* = 210)**	
≤33 weeks’ gestation	76 (36%)
34 weeks’ gestation	90 (43%)
35 weeks’ gestation	18 (9%)
Via cues of feeding readiness	148 (70%)
IDFS: Infant-Driven Feeding Scale	

**Table 2:** Survey responses for cold milk feeding practices.

Cold Milk Feeding	N (%)
**Are you aware of the practice of using cold milk for oral feedings? (*n* = 210)**	
Yes	64 (30%)
No	144 (69%)
No response	2 (1%)
**If yes, does your institution allow cold milk feeding for infants with dysphagia? (*n* = 64)**	
Yes	32 (50%)
No	31 (48%)
No response	1 (2%)
**If yes, at what corrected gestational age would you consider cold milk feeding? (*n* = 32)**	
>32 weeks’ gestation	2 (6%)
>33 weeks’ gestation	0 (0%)
>34 weeks’ gestation	5 (16%)
>35 weeks’ gestation	25 (78%)
**If yes to allowing cold milk feeding, how long have you been practicing cold milk? (*n* = 32)**	
<1 year	5 (16%)
1–2 years	6 (19%)
3 years	6 (19%)
4 years	1 (3%)
≥5 years	13 (40%)
No response	1 (3%)
**If yes to allowing cold milk, have you noticed any adverse effects with cold milk feeding? (*n* = 32)**	
Yes	8 (25%)
No	24 (75%)
**If yes to allowing cold milk, do you combine cold milk modification with thickened feeds? (*n* = 32)**	
Yes	9 (28%)
No	22 (69%)
No response	1 (3%)
**If yes to allowing cold milk, do you discharge the infants to continue cold milk feeding at home?** **(*n* = 32)**	
Yes	14 (44%)
No	18 (56%)
**If yes to allowing cold milk, do you have a protocol or guideline for when to initiate cold milk? (*n* = 32)**	
Yes	1 (3%)
No	31 (97%)

**Table 3:** Comments related to potential benefits and adverse effects of cold milk feeding.

Cold Milk Feeding Comments	
Comments About Potential Benefits	Comments About Potential Adverse Effects
“Improves quality of feeds.” “Improved engagement, decreased processing time, timeliness of swallow.” “Improved sensory awareness.” “Improved oral and pharyngeal awareness; decreased signs and symptoms of reflux; improved timing of swallow.” “Because of the better oral sensory input, the swallowing is stronger and more frequent. The flow of the milk is lower, and the baby has more time to swallow.”	“GI discomfort.” “Straining.” “Infant refusal.” “Some infants need temperature modulation, as the cold milk directly from the refrigerator can be too much sensory input, leading to pulling away from the feeding source.” “Intolerance.” “Hypothermia.”

**Table 4:** Survey responses for oral feeding on nCPAP and HFNC respiratory support.

Oral Feeding on Respiratory Support	N (%)
**Does your institution allow preterm infants to orally feed on nCPAP? (** ***n* = 210)**	
Yes	26 (12.4%)
No	183 (87.1%)
No Response	1 (0.5%)
**If yes, what level of nCPAP? (check all that apply) (** ***n* = 26)**	
nCPAP +3	11 (42%)
nCPAP +4	10 (38%)
nCPAP +5	16 (62%)
nCPAP ≥ +6	7 (27%)
**Does your institution allow preterm infants to orally feed on HFNC? (** ***n* = 210)**	
Yes	164 (78%)
No	44 (21%)
No response	2 (1%)
**If yes, at what flow rate can oral feeding occur? (check all that apply) (** ***n* = 164)**	
≤2 L	143 (87%)
3 L	55 (34%)
4 L	30 (18%)
>4 L	15 (9%)

**Table 5:** Comments related to potential benefits and adverse effects of feeding on respiratory support.

Feeding on Respiratory Support Comments	
Concerns About Feeding on nCPAP	Concerns About Feeding on HFNC
“Fear of aspiration.” “Aspiration, choking, and desaturation during oral feeding.” “If they are on CPAP, lung disease is severe enough to pose an aspiration risk.”	“Aspiration.” “Lack of evidence base to support that this is safe/positive.” “Need to use 2 litter/min or less.” “If they are on high flow, then lung disease is severe enough to pose an aspiration risk.” “Too high of a flow causing poor coordination.”

## Data Availability

All data generated or analyzed during this study are included in this article. Further inquiries can be directed to the corresponding author, Nazeeh Hanna (nazeeh.hanna@nyulangone.org).

## Supplementary Materials

The following supporting information can be downloaded at https://www.mdpi.com/article/10.3390/nu17142289/s1, Figure S1: Image of the survey questions via REDCap; Figure S2: A visual representation of the world map highlighting countries that participated in the survey.

## Abbreviations

The following abbreviations are used in this manuscript:

NICU	Neonatal intensive care unit
nCPAP	Nasal continuous positive airway pressure
HFNC	High-flow nasal cannula
PMA	Postmenstrual age
SoNPM	Section on Neonatal-Perinatal Medicine
NYU	New York University
ASHA	American Speech-Language-Hearing Association
NANT	National Association of Neonatal Therapists
SENTAC	Society for Ear Nose and Throat Advancement in Children
AOTA	American Occupational Therapy Association
SLP	Speech–language pathologist
PT	Physical therapist
OT	Occupational therapist
PIBBS	Preterm Infant Breastfeeding Behavior Scale
SOFFI	Supporting Oral Feeding in Fragile Infants
