# Supplementary material for: Oral Feeding of NICU Infants: A Global Survey of Current Practices and the Potential of Cold Milk Feeding Intervention
Source: Nutrients. 2025 Jul 10;17(14):2289. doi: 10.3390/nu17142289 (PMC12298771; doi:10.3390/nu17142289)
Supplement: Supplementary file 1 [file nutrients-17-02289-s001.zip › nutrients-3667811-supplementary.pdf]

Confidential

Page 1

Oral Feeding Survey

Dear colleagues in neonatal care,

You are being asked to participate in this study to help us understand variations in clinical practice of oral feeding in our preterm infant population. This survey has been approved by the Institutional Review Board at NYU Langone Hospitals. This survey is being conducted on behalf of the Dysphagia Collaborative Team at NYU Langone - Long Island by Drs. Nazeeh Hanna, Louisa Ferrera, Ranjith Kamity, and Zeyar Htun.

As this is a research study and participation is completely voluntary, by completing the survey you imply consent to participate in the study. Once the survey has been submitted you will be unable to withdraw from the study. The results of this study intend to be published in a peer reviewed journal, all data will be deidentified, and no identifiable information from the survey will be published. We are happy to share the results of the survey and our feeding protocols with you. If you wish to receive them, please click on the link at the end of the survey to provide us with your email addresses.

We would greatly appreciate you completing this survey which should take less than 10 minutes. Thank you for your time.

Prior to beginning the survey, please view the detailed information about the survey pertaining to purpose of the study, risk and benefits, and IRB information, please click on the PDF link. Afterwards, please proceed to the first survey question. Thank you.

[Attachment: "key-information-s2300109_V3.pdf"]

Question	Response
What is your current role?	◯ Neonatal Attending<br>◯ NICU Fellow/Advance Practice Provider<br>◯ Neonatal Therapist (SLP, OT, PT)
Where is your NICU located (City, State, Country)?	__________________________________
What is the level of your unit?	◯ Level II: Special care nursery<br>◯ Level III: Neonatal intensive care unit (NICU)<br>◯ Level IV: Regional neonatal intensive-care unit (regional NICU)
Do you have a feeding specialist (such as SLP, OT, PT) available in your institution?	◯ Yes<br>◯ No
Does your institution currently have an oral feeding protocol for preterm infants?	◯ Yes<br>◯ No
What protocol do you follow (i.e Infant-Driven Feeding Scale, cue-based feeding, etc)?	__________________________________
At what corrected gestational age do the preterm infants begin oral feeding in your unit? (may select more than 1)	☐ ≤ 33 weeks' gestation<br>☐ 34 weeks' gestation<br>☐ 35 weeks' gestation<br>☐ Via cues of feeding readiness
Do preterm infants receive oro-motor stimulation in your institution prior to initiating oral feeding?	◯ Yes<br>◯ No
Does your institution allow preterm infants to orally feed on CPAP?	◯ Yes<br>◯ No

05/12/2025 2:09pm projectredcap.org REDCap

Confidential

Page 2

Question	Response
If yes, what level PEEP can oral feeding occur? (check all that applies)	☐ +3<br>☐ +4<br>☐ +5<br>☐ ≥ +6
If no, what are the concerns in regard to infants orally feeding on CPAP?	__________________________________
Does your institution allow preterm infants to orally feed on high flow nasal cannula?	◯ Yes<br>◯ No
If yes, at what flow rate can oral feeding occur? (check all that applies)	☐ ≤ 2 liters<br>☐ 3 liters<br>☐ 4 liters<br>☐ >4 Liters
If no, what are the concern in regards to infants orally feeding on high flow nasal cannula?	__________________________________
Are you aware of the practice of using cold milk for oral feedings?	◯ Yes<br>◯ No
Does your institution allow cold milk feeding practice for infants with dysphagia?	◯ Yes<br>◯ No
At what corrected gestational age would you consider cold milk feeding?	◯ >32 weeks' gestation<br>◯ >33 weeks' gestation<br>◯ >34 weeks' gestation<br>◯ >35 weeks' gestation
How long have your institution been practicing cold milk feeding?	◯ < 1 year<br>◯ 1-2 years<br>◯ 3 years<br>◯ 4 years<br>◯ >5 years
What benefits have you witness with cold milk feeding?	__________________________________
Have you noticed any adverse events with cold milk feeding?	◯ Yes<br>◯ No
What adverse events have you witnessed with cold milk feeding?	__________________________________
Do you combine cold milk modification with thickened feeds?	◯ Yes<br>◯ No
Do you discharge the infants to continue cold milk feeding at home?	◯ Yes<br>◯ No
Do you have a protocol or guideline for when to initiate cold milk feeding?	◯ Yes<br>◯ No

05/12/2025 2:09pm projectredcap.org REDCap

Figure S1. Sample image of the survey questions asked on REDCap.

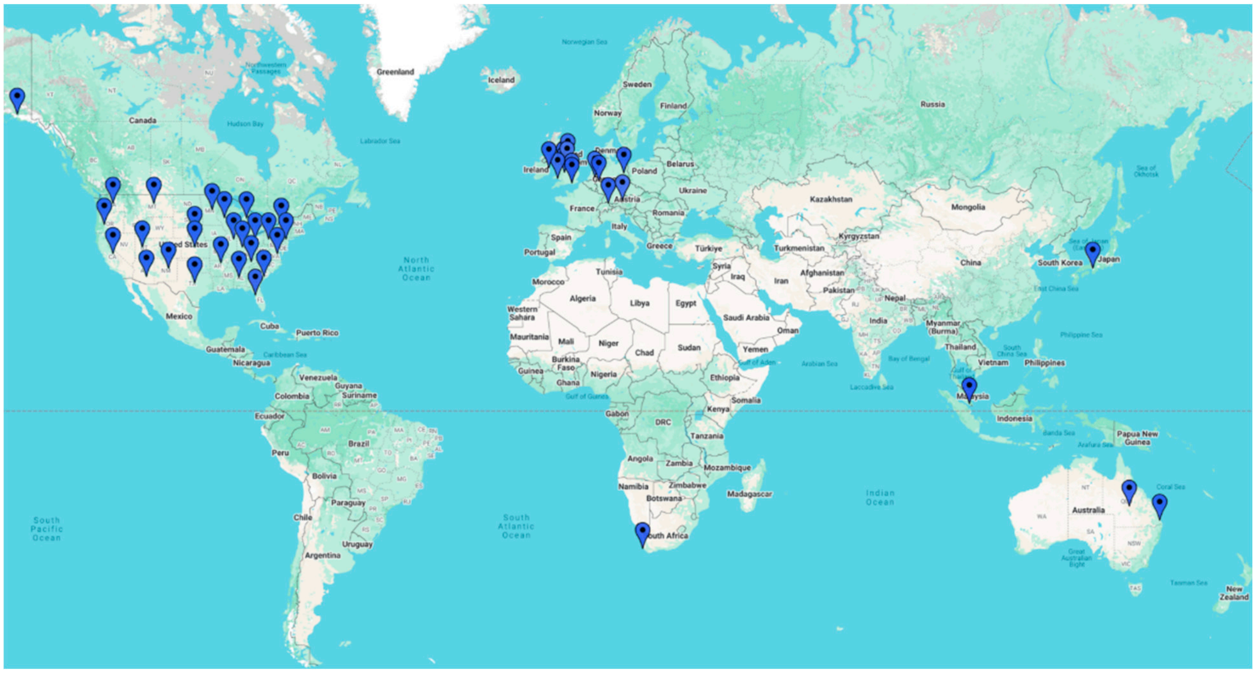

Figure S2. Distribution of survey worldwide. Markers show the geographical locations of the NICU of the survey respondents.
